# Supplementary material for: Shifting elder-care practices in Chinese middle-class families
Source: PLoS One. 2023 Mar 24;18(3):e0283533. doi: 10.1371/journal.pone.0283533 (PMC10038285; doi:10.1371/journal.pone.0283533)
Supplement: S1 Appendix — (PDF) [file pone.0283533.s001.pdf]

Appendix E: Everyday diaries, examples

The researcher wrote daily study diaries to record participants' routines after interviews. For example, in the Ye family, Mrs Ye (G2-D1) asked the researcher to visit her parents' home with her. In detail, the author recorded the following.

Table A.1: Family timetable for Ye family from author's observation

Time period	Daily practices
9am–9.45am	From G2 to G1's home by bus or being driven by her husband.
9.45–10.15am	Chat with helper: during the night Mrs. Ye's little sister (G2-D3) looks after their parents, so the helper took over duty at about 8am, then told Mrs. Ye (G2-D1) what was happening.
10.15–10.30am	Snack for G1-father, helping mother to the toilet (helper and G2-Mrs.Ye).
10.30–11.30am	Helper looked after older parents and Mrs. Ye guided her or gave her some support, such as water for toileting, cutting or washing hair and massaging, etc.
About 11.45am	Lunch is delivered around 12pm from institution to their home.
12–2pm	Lunch was delivered by institutional staff which include helper and older couple's meal, but Mrs. Ye (G2) shared the lunch with her parents (G1). Because the lunch was prepared for two people, Mrs. G1 ate very little.<br>Had lunch and had a little rest.

Similarly, in the Wang family, Mr. Wang (G2-S1) described their family practice to the researcher. But it was delivered by the domestic helper whom G2 had hired.

Table A.2: Wang family practice on eldercare

Timetable	Tasks
6am~	Mrs. G1 wakes up herself, carer helps G1 to wash
7am~	Prepare breakfast, help G1 to take medicines
9~10am	Clean the kitchen and rooms
10–11am	Have a rest
12pm	Lunchtime
1–3pm	Have a rest
4–5pm	Go to market or have a break
5pm	Prepare dinner
6–9pm	Prepare G1 for bed and chat
During night	Need to get up twice to help

Another example is that some interviewees were willing to share their family situations with the researcher. For example, there were family trees handwritten by participants.

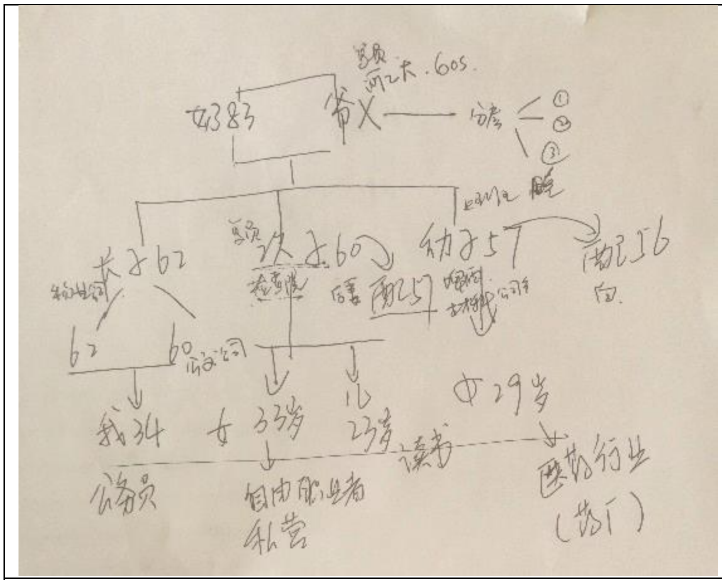	One-page note to show the example that interviewee made to researcher:<br><br>G3-Mrs. Xing drew their family tree and G2-Mr. Xing explained their family practices to the researcher<br><br>G3- Mrs. Xing introduced their family situations to the researcher and drew it out on paper
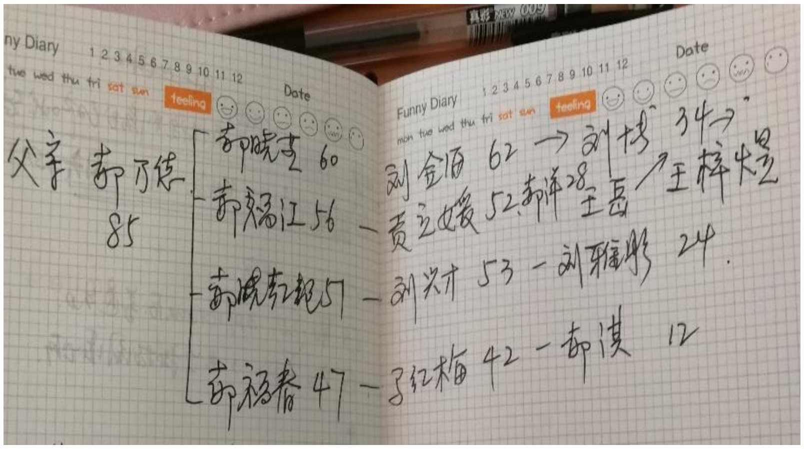	G2-Mrs. Hao drew their family tree and spoke about their family members' situations

<u>Appendix F: A summary of each action that the researcher carried out in the field</u>

In total, ten families were visited and interviewed during this fieldwork, including seven families with three generations, and three families with two generations. The table below shows the four focus groups, and the volunteer group of retirement people from Hebei District, Tianjin, which the researcher joined. Moreover, the researcher also visited institutions that supported different groups.

Fieldwork type	Date	Photos	Participants	Comments
Focus group 1	08-01-19	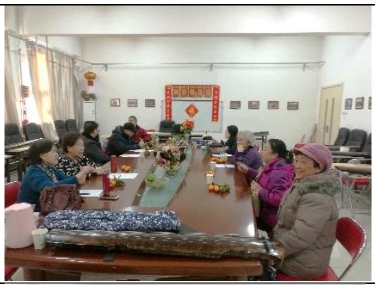	8 persons	Neighbours and friends of my parents who were contacted to join a focus group on ageing-related research
Focus group 2	09-01-19	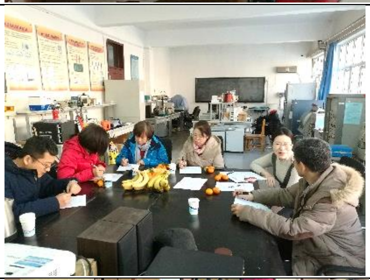	6 persons	My father's friends: he helped me get in touch with the participants. In their office, I conducted a focus group
Focus group 3	04-04-19	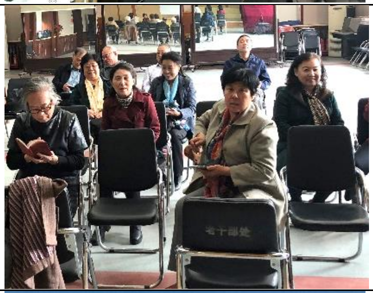	6 persons	After a singing lesson, a discussion group was held to recruit more interviewees
Focus group 4	Photo taken on 15-05-19; between March and May, I joined the Tai Chi team.	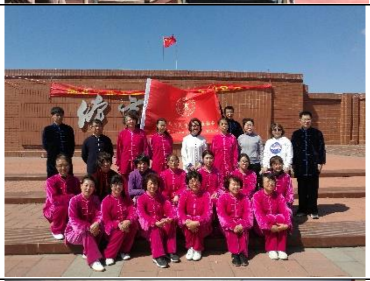	8 persons	Informal focus group (discussing the expectations of children taking on the obligations of eldercare)
Older people's volunteer team	12-01-19; 19-01-19	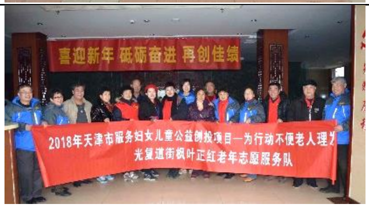	Older people's volunteer team that has been running for six years	Helping older people in institutions with cutting hair; notice the volunteer team from website on internet

Visit to Song He institution (Community service)	22-02-19	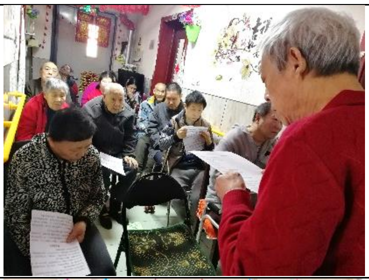	With Mrs. Ye; deliver help to home.	This was the day that G2-Mrs. Ye was signing the contract with the care home. The manager invited me to observe
Visit to Jinghai institution (Service to Jinghai district)	05-04-19	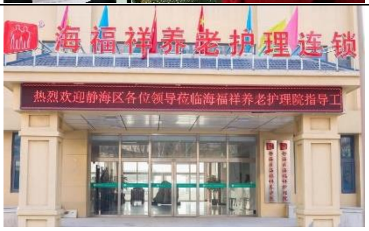	5800 (CNY) per person including bed and all bills.	A chain institution was supported by local government. An interviewee was considering it and asked me to visit
Visit to Jingwan elderly people's home (service to Tianjin, Beijing)	15-06-19	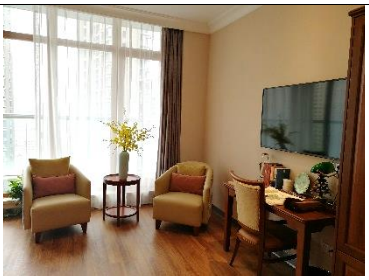	6000 (CNY) per bed; Caregiver fee: 500–9,000; meals: 2000 per person; total: 8,500–17,000 per person	Developer is Yuanyang real estate, which is a high-quality and city-centre location institution
G2-Mrs. Ye and Mr. Zhang	17-01-19; 19~24-02-19; 26~28-02-19; 21-06-19	Their home-G2; Parents' home-G1; Via WeChat chat to G3		Took notes, photos; interviews with G1-G2-G3
G2-Mrs. Hao's family	04-03-19; 18-03-19; 25-03-19; 16-06-19	Yicheng tingtang-G2 home; Qingchun nanli-G1/G3 home	Questionnaire	Took notes, photos; interviews with G1-G2-G3
G2-Mr. Li & Mrs. Li	01-02-19; 06/13/20-03-19; 03-04-19; 21-06-19	Their home-G2 home; Litai community-G1 home; Fuyuan community-G3 home	Questionnaire	Looked at their holiday photos; took notes, photos; interviews with G1-G2-G3
G3-Mrs. Xing	26-01-19; 28-02-2019; 24-03-19	Shopping mall. G3-Xingjing's home (*lingshi soho*); Anyue li-G2/G1 home		Took notes, photos; interviews with G2-G3
G2-Mrs. Dai and Mr. Wang's family	05~12-02-19; 10-04-19; 06/09-06-19	Teacher's community in Hebei University of Technology (G2 home), Tianjin. Baihua community (G1 home), Shenyang. G3-rented a room in Beijing	Questionnaire	Took notes, photos; interviews with G1-G2-G3

G2-Mrs. Zhao and Mr. Jia	10-01-19; 16-02-19; 13-06-19	Their home-G2; talked to G1 via WeChat	Questionnaire	Took notes, photos; interviews with G1-G2-G3
G2-Mrs.Xu and Mr. Huo	14-02-19; 06-05-19; 20-06-19	Their home-G2		Took notes, photos; interviews with G1-G2-G3
G3-Mrs. Kong song	08-01-19; 27-02-19	Shopping mall. her home		Took notes, photos; interviews with G2-G3
Mrs. Fu & Mr. Wang	28-03-19; 29-03-19; 30-03-19	Hai er dong cheng guo ji (海尔东城国际)， Qingdao-G2/G3 home; Teacher’s community in Hebei University of Technology (G2 home), Tianjin		Migrated to Qingdao from Tianjin (two adult generations)
G3-Mrs. Dong Jun	03-02-19; 10-04-19; 21-04-19	Her office. her home		Took notes, photos; interviews with G2-G3
