# Supplementary material for: Shifting elder-care practices in Chinese middle-class families
Source: PLoS One. 2023 Mar 24;18(3):e0283533. doi: 10.1371/journal.pone.0283533 (PMC10038285; doi:10.1371/journal.pone.0283533)
Supplement: S2 Appendix — (DOCX) [file pone.0283533.s002.docx]

# Appendix 1: Samples of interview questions

The researcher had planned to ask interviewees some questions about the themes below.

**Start questions:**

Since when have you lived in this community? How do you feel about living in this community?

Could you please draw your family tree for me? Have you or your parents retired? When?

**Theme 1**: what the later life means to you (G2 older parents and G1 grandparents)

1. What would you imagine in a good later life?

2. Who would you like to live with and why?

3. What would you think the most important factor is that influences your lifestyle in later life? For what purpose do you care for yourself?

4. If you had the last chance to move home, where would you like to live? Why?

**Theme 2**: relationships within generations (G3 adult children or G2 older parents)

1. Who takes care of older family members?

2. How will older people live a good life in later life?

3. How will they source care if needed?

4. How do you feel about filial piety? Do you think that you fulfil filial duties?

5. How often do you visit your parents?

6. Do you have any experiences of facing multiple transitions, such as retirement, illness of your spouse, the birth of your (grand)children, or something else? Did the emergency situations happen at the same time? How could you manage that situation? How could you balance that?

7. Imagine one of your parents becomes frail, or you become frail or you need long-term care; how would you manage it?

8. If an emergency happens, whom would you want to contact first of all? Why?

**Theme 3**: interviewing generations together: if one family member needs care, could you tell me how resources flow in this table? Or if it happened previously, how did your family manage that?

• Picture 1: key words ‘people resources flow’

• Picture 2: key words ‘how practical care flow’

• Picture 3: key words ‘space, house and move’

• Picture 4: key words ‘time’

• Picture 5: money

Grandparents (pension, welfare benefit per month)

Older parents (may or may not have income, medical insurance)

Adult children (income)

• Picture 6: know-how

| Generations | G1:  Grandparents  around 80s | G2: Older parents 55–79  (Sandwiched generation) | G3: Young adults  30s–40s | G4: Young  children |
| --- | --- | --- | --- | --- |
| Know-how  (Information  resources) | Newspaper,  TV? Relatives?  Friends?  Children? | Social media, TV,  newspaper? Neighbours?  Ex-colleagues?  Friends? | Social media, online  news? Friends?  colleagues？ | Online?  Classmates?  Parents?  Relatives? |

**Follow-up questions**:

How does care flow across the family?

How do people shift their expectations on care?

What is the way that people use time and money?

Where are the conflicts in your family?

**Values, views and feelings**:

How did you respond when…?

What did you feel when…?

Why did you think it was important to you?

**Exploring impacts, effects and consequences**:

What effect did that have on you? Did that help you in any way?

How did your approach change when you found that out?[31] (pp. 150-151).
