# Supplementary material for: Shifting elder-care practices in Chinese middle-class families
Source: PLoS One. 2023 Mar 24;18(3):e0283533. doi: 10.1371/journal.pone.0283533 (PMC10038285; doi:10.1371/journal.pone.0283533)
Supplement: S1 File — (DOCX) [file pone.0283533.s003.docx]

# Chapter 5: Background of the city and the families

Chapter 5

Contents

## Introduction

This chapter acts as a preface to the next three field chapters that explore interesting narratives and the pressure of family practices on the care of older adults. This chapter’s first section allows readers to identify the history, location, and the standard of living in the city of Tianjin. The second section addresses the background and family relationships of interviewees within middle-class households in Tianjin.

## City of Tianjin

### History and development

Tianjin, is a port city and coastal city that opened to the outside world during the Qing dynasty (1636–1912), from which it has maintained its position as the economic centre of northern China. After the founding of the People’s Republic of China in 1949, together with Beijing and Shanghai, it was identified as one of the first batch of cities to gain a municipality (the city under the jurisdiction of the central government). Until 2006, the State Council reviewed and approved the *Tianjin City Master Plan (2005-2020)*, which clearly positions Tianjin as an international port city, a northern economic centre and an ecological city. However, Tianjin’s economic performance has deteriorated in recent years, resulting in its downgrading to a tier-two city. Only nine years later, the Central Leader Group adopted and launched a new plan to integrate the resources of Beijing, Tianjin and Hebei and coordinate development, which meant that a new plan was developed which would focus on regional cooperation and reallocation of resources (Liu and Cao, 2017). Tianjin is the second-largest megacity after Beijing in the Jing (Beijing)-Jin (Tianjin)-Ji (Hebei) region (*Xin Hua News*, 2019); the city is adjacent to Bohai Bay (see Figure 5.1).


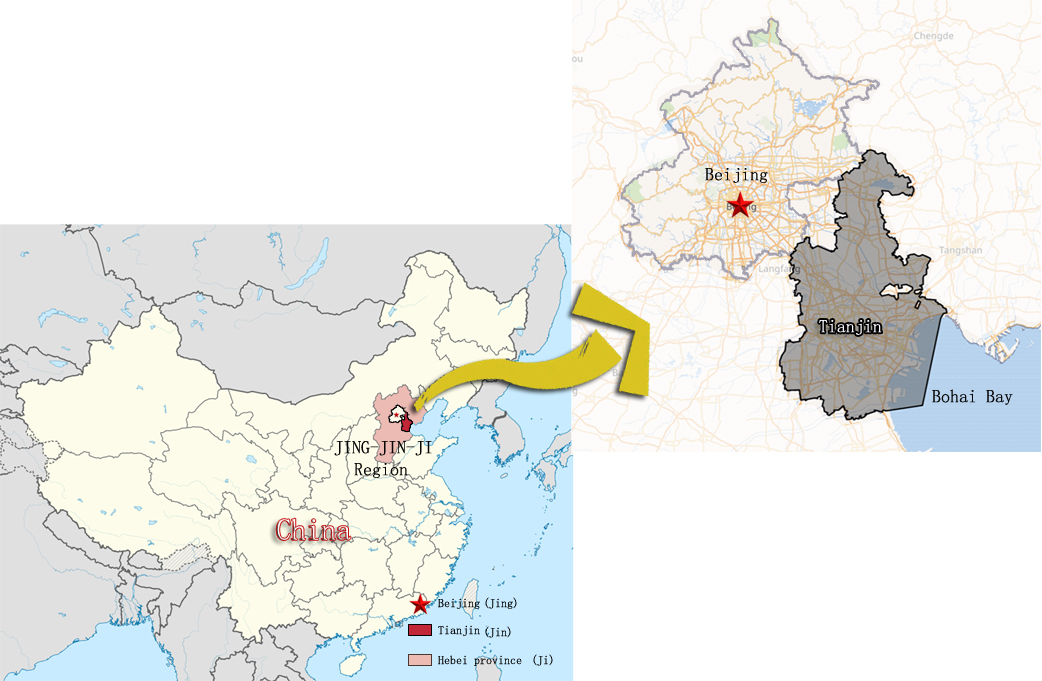


Figure 0.1: Jing (Beijing)-Jin (Tianjin)-Ji (Hebei) region and location of Tianjin

Tianjin has been well known as a major port next to Bohai Bay since the Sui dynasty (581–618 ce) and the development of the Grand Canal in China. The urban plan is influenced by the direction of the *Haihe* river. Urban construction was influenced by Western-style types of building with the signing of the Treaties of Tianjin which opened the city to foreign trade from 1858. Due to the importance of its location, Tianjin became a vital port that connected the northern and southern parts of China and was an important node of communication between neighbouring countries. As a result, Tianjin has become a world-famous port city with a throughput of more than 100 million tons, dozens of routes, and trade connections with 168 countries in 2004 (Tianjin Local History Compilation Committee Office, 2005).

Tianjin has a total population of 15.59 million (Tianjin Statistical Bureau, 2019). The non-resident population – migrant workers – accounts for 32% of the permanent population, which is 4.99 million (Tianjin Statistical Bureau, 2019; HKTDC Research, 2020). The older adults (age 60 and over) reached a total of 2.59 million, which accounts for 23.97% of the total population (Tianjin Civil Affairs Bureau, 2019). Tianjin focuses on the secondary and tertiary industries, accounting for more than 99% of the total industrial production of the city (Tianjin Statistical Bureau, 2019). For instance, Tianjin was one of China’s earliest pilot bases for the circular economy, a national-level neo-industrialization base for electronics, automotive and petrochemicals, and a national-level innovation and entrepreneurship base for senior talent returning from overseas (Deloitte Global, 2017).


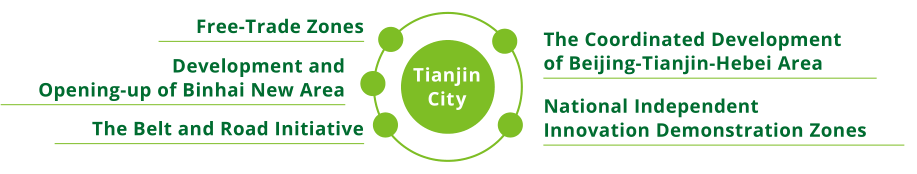


Figure 5.2 Tianjin city’s industry plans (resource from Deloitte Global 2017)

By virtue of its proximity to the capital, Tianjin is a popular choice for industries wanting to relocate from Beijing, offering a lower level of required investment in land or buildings, a pleasanter environment and easier transport. Since 2014, Beijing has planned to further develop its focus on political and cultural functions, which is an increasing trend (Liu and Cao, 2017). The high-speed railway development of 2008 means that travel time between Beijing and Tianjin has been cut from two hours to around 30 minutes (Liu and Cao, 2017). Also, the (Tianjin) Pilot Free Trade Zone (TJFTZ) project was officially launched in April 2015. As the first free trade zone in northern China, Tianjin’s strategic position will be linked to the coordinated development of Beijing, Tianjin, and Hebei (Tianjin Local History Revision Committee, 2015). This plan may be traced back to 1980, when the economic reform policies of Deng Xiaoping took shape. Indeed, during these early years, it was understood that Tianjin would become a free trade zone like Hong Kong.

Tianjin has a continental climate, which means that it is cold but has sunny winters, and hot, sultry and rainy summers. According to the Economist Intelligence Unit’s rating, Tianjin has been described as being a ‘liveable’^[[1]](#footnote-1)^ city, ranking third in mainland China (77th in the world), following behind Suzhou (ranking first) and Beijing (ranking second) (Feng, 2018). The list of global liveable cities was assessed using five aspects: stability, healthcare, culture and environment, education, and infrastructure. After all, Tianjin is a comfortable coastal city, which has a concentration of middle-class families and whose population of older people is nearly one-quarter of the total.

### Life in Tianjin

Compared with tier-one cities (Beijing, Shanghai, Guangzhou, Shenzhen), Tianjin is a less economically stressful city. Among other tier-two cities, it has the highest ratio of housing prices to income in that it is higher than 60% (up to 63.3%) (Mu, 2020). The stress on people of dealing with affordability or pressure in housing markets to deliver more is dramatically high.

There are mainly four types of property in the urban area, including *lao-po-xiao* (old-poor-small) communities, ungated communities (work unit properties), commercial communities (similar to gated communities) and luxury villas.

*Table 0.1: Community types in Tianjin*

| Community type | Mode of governance | Residents | Brief description |
| --- | --- | --- | --- |
| *Lao-po-xiao* community;  old community built before 2000 | Administered by lowest governmental organisation, such as street committee | Working class, lower- middle class | Ready to be demolished in three years; located in the inner-city area; low quality of facilities |
| Ungated community (work unit) | Welfare-style governance | Lower- middle class | Normally built in the 1980s–90s |
| Gated community (private-sector development) | Ownership-based governance | New or middle-middle class | More green spaces, better facilities and parking places, etc. |
| Luxury villa | Collective-based governance | Upper-middle class | Normally a holiday house located on the edge of the urban area |

Source:(Breitung, 2012)

Interviewees in this study were mainly concentrated in the ungated and gated (private-sector development) community. The teachers’ community is a kind of work unit apartment is only sold to the teachers as employees. It is easy to draw comparisons of gated and ungated (former work unit) communities, as the latter has a close social network, while the new commercial community is developed by real-estate companies, where the residents are living with people unfamiliar to them; but each has similar social status, a good school district and convenient service resources.

The location is an important factor that influences the price of housing (see Figure 5.2). Higher-priced properties are closer to the city centre, and the highest prices are in Heping district because of the best education resources. According to the prices in six districts in Tianjin, a three-room apartment (about 100 m^2^) costs around 2.4 million CNY^[[2]](#footnote-2)^ (about £275,068) to 5 million CNY (about £573,057). Considering the price of properties are continually increasing; it is understandable that middle-class households take their investment in the real-estate market seriously.


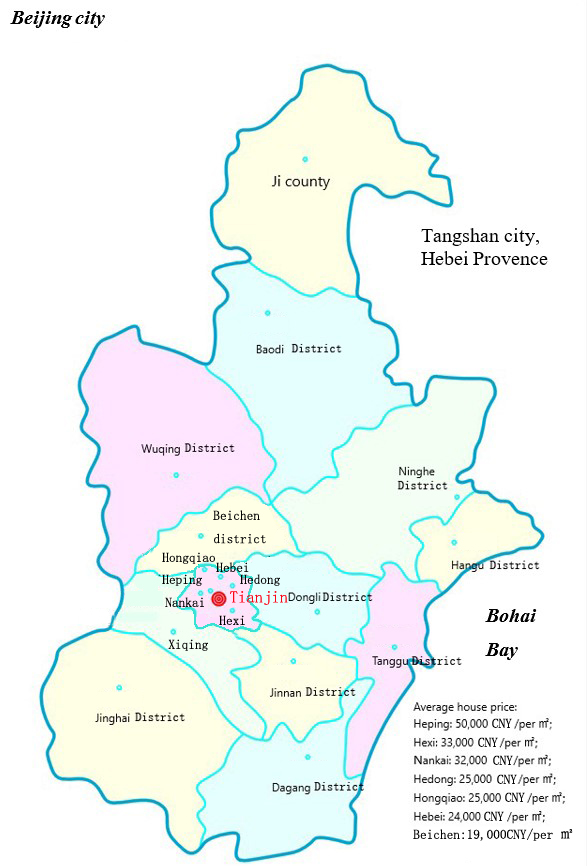


Figure 0.2: House prices in different districts in Tianjin ([www.LianJia.com](http://www.LianJia.com))

During the interviews, it was evident that people’s material life, for example their income and living conditions, had changed dramatically over three generations. Following the economic reforms post-1978, the real-estate market experienced high-speed development, and Tianjin, as a tier-two city, was no exception. Co-residence and extended families, once a normative situation, is no longer common or seen as valued by multiple generations, which results in more complex situations in caring for older people and children.

### Increasing number of older people and responses

Tianjin has 2.59 million older adults, which accounts for 23.97% of its total population (Civil Affairs Bureau, 2019). In the end of 2014, the local government of Tianjin launched a policy on ageing. This policy, named ‘Accelerating the construction of elderly services in Tianjin’ (*Tianjin shi yanglao fuwu cujin tiaoli*), set out to integrate community-based and institution-based services (Tianjin Civil Affairs Bureau, 2018). In 2016, the Tianjin government also reported on the pension situation of the city, which already covered more than 80% of older people (Tianjin Municipal People's Government, 2016). At the end of 2018, there was a total of 1301 day care centres serving older people and 10,000 beds in care homes (Tianjin Statistical Bureau, 2019). According to the standard of 30 beds per thousand older people in care homes, there is a shortfall of 11,000 care beds for frail older people, and the provision only covers 24% of communities (Tianjin Civil Affairs Bureau, 2019). The rising number of older people has prompted the local government to act, but arguably more reforms are needed. The table (5.2) below provides information on private institutions in the Jing-Jin-Ji area. It has been shown that beds for the seniors are already in short supply though, private aged care institutions have a relatively low occupancy rate.

*Table 0.2: General information of private seniors’ homes in the Jing-Jin-Ji region*

| Area | Project | Location | Management mode | Up-to-date sale situation |
| --- | --- | --- | --- | --- |
| Beijing | Tai shen xiang he (太申祥和) | Beiqing Road, Changping District, Beijing | Operating on a business (membership fee: 200–500,000 CNY; first three years free from monthly fee; catering and medical fee not included) | Started in 2002, a total of more than 1000 members, more than 500 permanent members. First membership care home in China. |
|  | Yao yang guo ji (曜阳国际) | Xi Tian Ge Zhuang Town, Miyun County, Beijing | Apartments for sale and rent. For sale: 14,000 CNY/m^2^; for rent: 58,000 CNY/year (catering fee not included) | Started on site in October 2010, and sold out in April 2011. |
|  | Dong fang tai yang cheng (东方太阳城) | Chao Bai river bridge, Shunyi District, Beijing | Apartments for sale (16,000 CNY/m^2^) | Between 2008 and 2012, first and second parts were sold out; third part on sale includes: 2000 apartments at 16,000 CNY/m^2^, 125 villas at 30,000 CNY/m^2^, but they have not sold well. |
| Tianjin | Bin hai yun shan zhen (滨海云杉镇) | Niu Kou Dao Town, Baodi District, Tianjin | For sale and rent. For sale: around 1,000,000 CNY for one apartment; for rent: 30,000 CNY/year (not including catering and medical fees) | Started in 2012; signed-up membership only 50, considering that there is space for about 300 persons. |
|  | Yong tai hong kan (永泰红磡) | Li Shuang Road, Jinnan District, Tianjin | Holding business (annual fee: 60,000 CNY) | Have not sold well. |
| Hebei | Tai da guo ji jian kang cheng (燕达国际健康城) | Yanjiao high tech industrial development zone | For sale and rent. For sale: 1,680,000 CNY/ apartment; for rent: from 8,000 CNY/month | Started in 2010; residential rate was 50%, until 2014, fallen to around 10%. Tenant moved out. |

Source: (Liu et al., 2018)

The average pension in Tianjin is 3032 CNY (about £340) per month and an older person with this pension cannot afford to live in these developments, demonstrating that they are geared to higher-income groups. However, even middle-class retirees, with higher pensions of perhaps more than £1000 a month, would find it difficult to afford to live in these private institutions. They prefer to see it as an investment to own another property, which means that they gather family members’ savings to buy that apartment, not to live in, but aiming to sell it for a better price or give it to their children.

## Interviews of families

In this study, ten families were recruited to the research to discuss their care practices. Details of these are presented and analysed in the three fieldwork chapters that follow. Here, each family is presented as a cast of characters who later tell their stories. Family names have been altered to preserve confidentiality.

The first group, in Chapter 6, are four families who take the traditional path of *relying on themselves*. These are the Li, Hao, Xing and Huo families. The second group, in Chapter 7, presents three families, Ye, Zhao and Wang, who choose to use *additional help*. This illustrates the situation of those families that cannot meet older members’ needs by themselves. There is a trend of market-based support being blended with family care. The third group, in Chapter 8, presents three families *whose G1 is no longer alive*, which means that those in G2 are the oldest members in their family. These families, the Kong, Han, and Fu families, are considering their future plans for ageing.

Each family is now presented in more detail, providing a statistical profile with an emphasis on education, income, and property. A family structure figure shows the relationships between family members. The blue line in each figure shows who has been interviewed in this study, and the red line shows the gatekeeper (the person in each family who was first approached and introduced me to the family). The generations are expressed as G1 (grandparents: mainly in their 80s), G2 (older parents: usually in their 50s–60s), G3 (adult children: around 30). D and S denote sons and daughters. In families with more than one child, each is given a numerical identifier according to their birth order. For example, the oldest daughter in the Li family in generation two is described as G2-D1, and her younger brother as G2-S2, and so on.

G2 can be described as being to the third age – early retirees in good health – in this study and G1 is of the fourth age, who is, in some extent, experiencing frailty and needs varying levels of support. This is a major discourse and symbolic indicator of the late-life boundaries between health and disability, and by implication, what is anticipated in relation to such a late-life agency (Baars and Dohmen, 2013, p. 57).

### Changes in the historical texture

Although lifestyle changes continually occur because of technological advances, historical events over the last century have impacted dramatically on people’s lives, creating great generational differences. Figure 5.3 shows the historical experiences in the early years of G1, G2 and G3’s life stories.


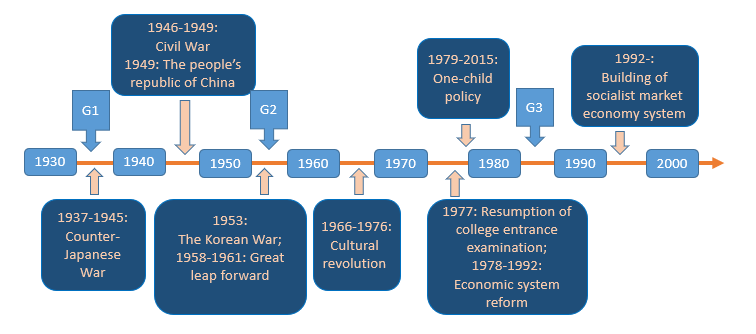


Figure 0.3: Timeline of historical events in China

The three generations belong to different historical cohorts. with very different memories and life stories. These three generations have experienced totally different social welfare policies, which result in totally different social norms, values and family structures (Xie, 2013). Until now, G1 has struggled with survival and making a living throughout most of their lifetime. G2 endured widespread hunger, insecurity and a lack of schooling during their early years. To earn a better standard of living than their parents, G2 had to rely on themselves. G3 was born in a comparatively harmonious, increasingly stable, and neo-liberal country, but they are the one-child generation, and potentially the only one-child generation, who, soon, will face the dilemma of taking care of their older parents, (see age range G1-G2-G3, table 5.3).

Table 5.3: Generation age range in this study (interviewee)

| Age range in the study | G1 | G2 | G3 |
| --- | --- | --- | --- |
| Year-old | 79-87 | 47-69 | 25-41 |
| Born-year | 1940-1932 | 1972-1950 | 1994-1978 |

The Confucian principle of filial piety is a strong link binding these three generations together, but in the Maoist era much of the care for older people and children was taken over by government and delivered through state-run work units. However, this was also a time when all three generations lived together, delivering inter-generational support. Another dramatic change occurred after the economic reforms (after 1979), when many state-run enterprises were failing and closed along with the clinics, nurseries and community support that they ran. Since then, several policies have been launched that place the responsibility of caring for older people (and young children) on families, reinvigorating the idea of filial piety. However, a lasting change is that gender equality in the labour market has become firmly rooted in China from the Maoist era onwards. Women’s social economic status may be enhanced but they are assumed to take on double duty, with work outside the home and care of the home and those within it.

Moreover, due to the one-child policy, the normal family size shrank. In November 2015, the government abolished the one-child policy, enabling women to have a second child without seeking permission; however, the fertility rate has not increased as expected (BBC NEWS, 2018). The country had 18.4 million births a year back, up 1.4 million from the five-year average. The government had predicted 4.3 million births, but the actual number was much lower. The government is facing challenges that is exemplified by its weak reaction to a historic policy reform (Kiprop, 2020). The generation of one-child parents are therefore positioned between their parents, with their potential care needs, and their children, and they will need support in the future when they grow up and have a family of their own.

### Families who *depend on themselves*

There are four families in this group: the Li, Hao, Xing and Huo families. They take the traditional path by relying on themselves.

#### The Li family

The Li family is made up of four generations: the G1 couple; three daughters in G2 and their husbands; three only children in G3; and two little girls in G4.


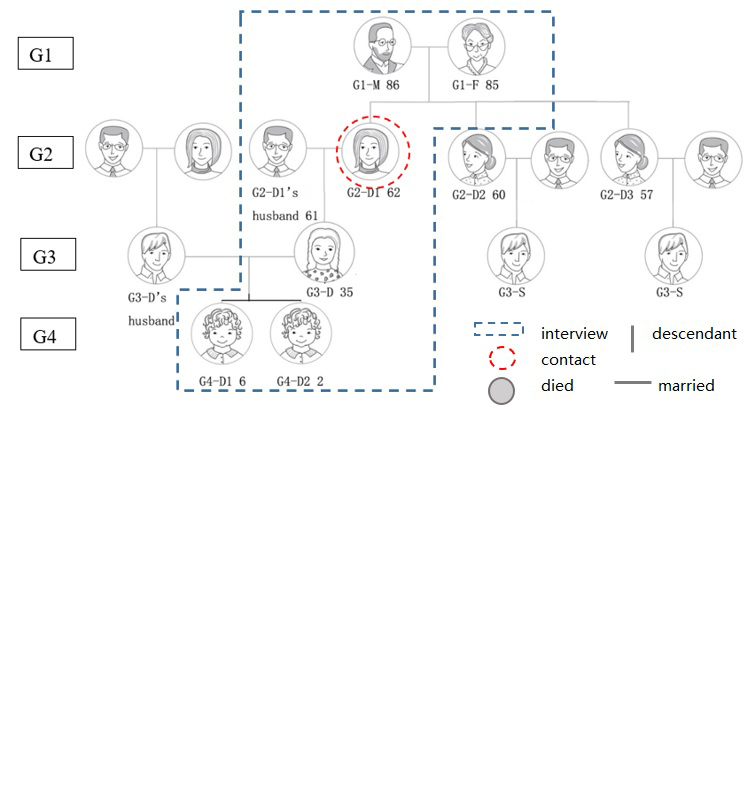


Figure 0.4: Li family

The interviews with the Li family focused primarily on the G2-D1 family, including the G1 couple, G2-D1 and her husband, as well as their daughter. G2-D2 sometimes also took part in our discussions. Their family tradition is that every Wednesday, G2-D1 and G2-D2 visit their parents; sometimes G2-D1’s husband comes with her to help, sometimes G3 (her daughter) takes her little daughters to visit G1 together.

The G1 couple are very healthy. Mr. G1 encountered difficult times both before and after the establishment of the People’s Republic of China. Though retired, he is still reading and writing, and he has written countless articles. He also contributed, as he said, to university administration, such as the School of Electrical Engineering, Humanities and Law. His writings show that he is a hard-working scholar. Mrs. G1, when she was a teenager, came to Tianjin from a south-eastern city with her older sister and brother. In her marriage, Mrs. G1 takes on all the responsibilities of the household. Up until now, she has been taking care of her husband and giving her granddaughter money.

G2 consists of three daughters. They all have an undergraduate degree, which for the people of their generation is rare. G3 consists of one female and two males. All the boys in G3 have worked overseas and now live in the United States. Except for them, all the Li family members live close by. This geographical proximity is also seen in other families in this category who depend on each other to take care of older family members.

#### The Hao family

The Hao family is made up of four generations: Mr. G1 (82 years old), two daughters and two sons in G2, and four only children in G3, with only one girl in G4.


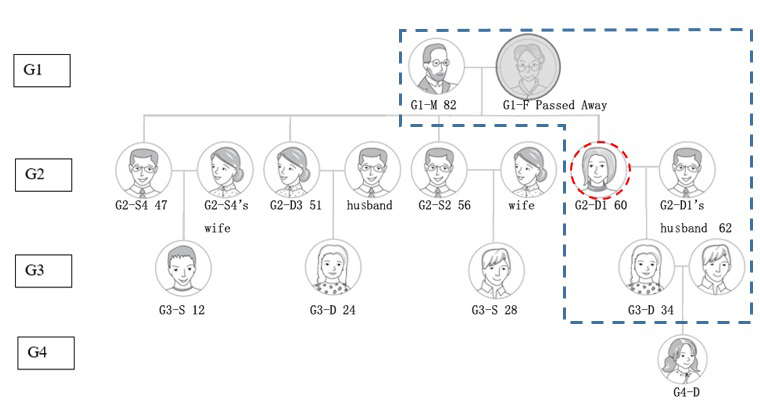


Figure 0.5: Hao family

The contact person in the Hao family is G2-D1, who was introduced by another participant. She hospitably invited the researcher to visit her home and actively participated in interviews. However, when the researcher asked to contact her husband and daughter, she politely refused. After several visits, G1, G2-D3 and G2-S4 were successfully interviewed, but G2-D1’s husband and daughter briefly filled in questionnaires.

Mrs. G1 passed away several years ago, and now Mr. G1 lives alone but he is in good health and seems younger than his age. He looks after himself, and his youngest son (G2-S4) visits him regularly. In the Hao family, most of G2 are working in the same university except for G2-S2, including G2-D1 and her husband, G2-D3, G2-S4 and his wife. G2-D3’s husband also works in an American university where he had obtained a doctorate. Only G2-S2 is the manager of a local factory, and he is chosen to obtain a government signing bonus because he has contributed to countless technical breakthroughs.

Two of the G3 (children of G2-S2 and G2-D3) work in the United States and are thinking about staying there. The daughter of G2-D1 already holds a master’s degree from a university in the United States and has returned to Tianjin.

#### The Xing family

The Xing family is made up of four generations: Mrs. Xing (83) in G1; three sons in G2; three only children in G3; and three girls in G4.


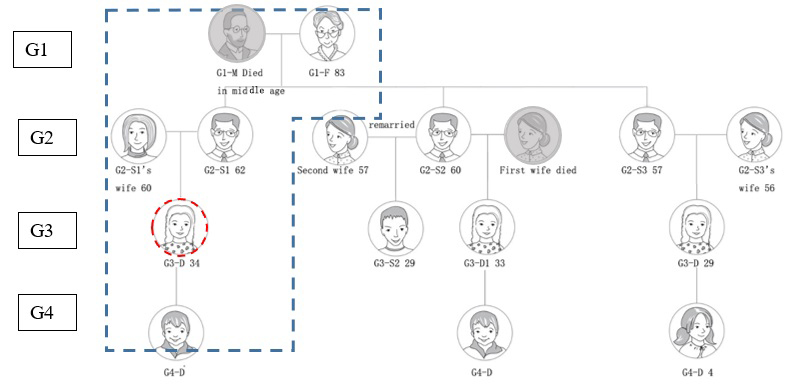


Figure 0.6: Xing family

The gatekeeper of the Xing family is one of the G3, who is the daughter of G2-S1. The conversations took place between the researcher and G2-S1, his wife and their daughter (G3) separately. Mrs. G1 has been suffering from dementia for more than five years. The researcher paid particular attention to the observation and interpretation of the care practices because of her health condition.

In the Xing family, the G1 couple were middle class, because Mr. G1 used to be the head of a school and owned two apartments, but he died when he was 50. Mrs. G1 (83) used to be a civil servant who worked in the street office (the lowest level of government in China) and she has a decent pension after she retired. However, most of G2 seemed to lose their middle-class status. Two sons in G2 were laid off and only G2-S2 kept his job and status as a prosecutor. G2-S2 is divorced from his first wife, who subsequently committed suicide. Afterwards he remarried and could have a second child.

Their frail mother (Mrs. G1) is looked after by her three sons in turn, but most practical support is arranged by the eldest son (G2-S1). In G3, only the daughter of G2-S1 graduated with a master’s degree and works in the education department as a civil servant. The other two girls (G3) are self-employed with high-school diplomas.

#### The Huo family

The Huo family is made up of three generations (see Figure 5.7): the G1 couple, two sons and one daughter in G2, and three only children in G3.


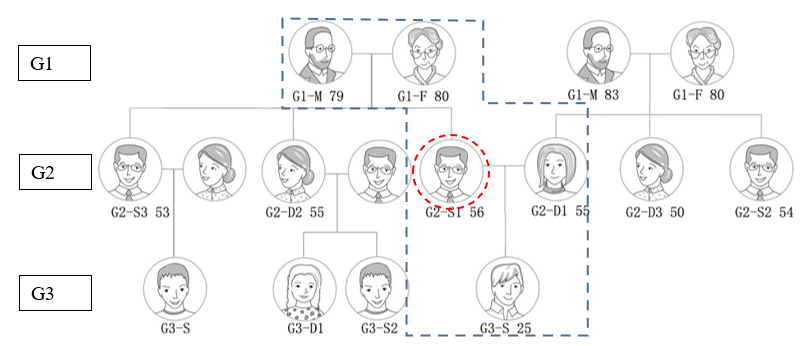


Figure 0.7: Huo family

G2-S1 was a participant in a focus group who was interested in taking further part in this study. In addition, his wife was also interested and helped the researcher to interview her parents by using a WeChat (a popular Chinese social app similar to WhatsApp) video call.

The Huo family is relatively younger because G2-S1 (56) still had four years left to reach the required retirement age of 60 in 2019. He has a hobby that already occupies all his spare time. His wife (55) found a part-time job for after her retirement, which would be in September 2019. She also has her activities, such as playing tennis and badminton.

The G1 couple in the Huo family are in good health. But they have no pension, because of Mr. G1’s investment losses some decades before. The daughter (G2-D2) married a man in the Tanggu district of Tianjin, but unfortunately, their child has a serious disease. Since then, the G1 couple has moved to help their daughter. They (the G1) live in another apartment that was bought by G2-S3, which is located close to G2-D2’s home. Now, it is also convenient for G2-D2 to visit her parents.

G2-S1’s wife also has two other siblings; they live in the same city, Xingtai (in Hebei province), as their parents (G1) do. These parents are obviously in a better financial situation than the G1 in the Huo family. Her father used to be a leader in the town, and he receives a pension of more than 8000 CNY (about £907) monthly.

To sum up, those families who wish to be self-dependent must meet some or all the following conditions. First, G1 must be in good general health, able to look after themselves, or are married and can take care of each other. Second, at least one child in G2 lives nearby, who can deliver support regularly. Third, their family relationships are good or the financial situation of G1 is relatively comfortable. However, if one of G1 is frail or bedridden, their family resources may be strained, and this may result in asking for additional help. The next section introduces three families who hire domestic helpers to take care of their older parents.

### Families who ask for additional help

#### The Ye family

The Ye family consists of three generations (see Figure 5.8): a G1 couple, three G2 daughters and three G3 grandchildren.


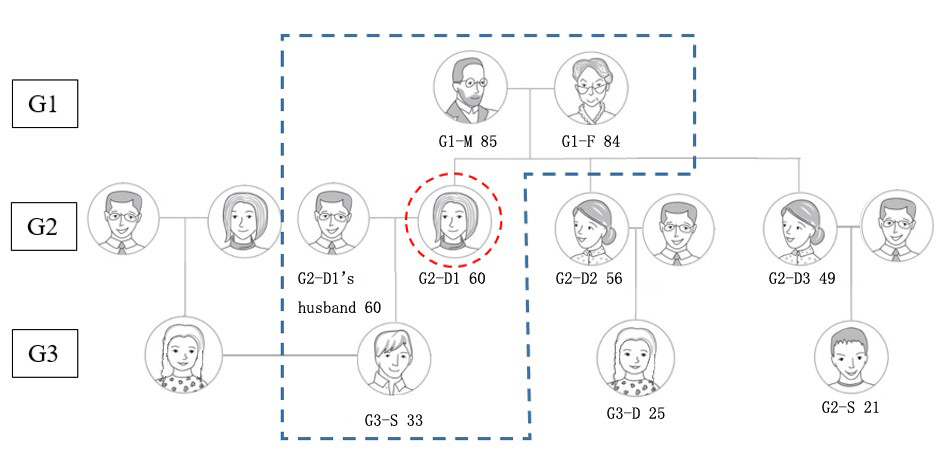


Figure 0.8: Ye family

The three sisters (G2) have lived in Tianjin since they were born and feel deeply rooted in the city. G2-D3’s husband and her son (G3) are working and studying in another city. The son (G3) of G2-D1 and his wife have settled down in the United States. Interview conversations were mostly with Mr. G1, G2-D1 and her husband, and their son (G3) who was interviewed by video call. In addition, G2-D2, G2-D3 and the helper were interviewed separately.

Although there are three daughters in G2, G2-D2 is disabled and cannot deliver physical support to their parents. Mrs. G1 is living with dementia and has been bedridden for five years, with her three daughters’ providing care. After Mr. G1 fell recently and suffered a fracture, they (G2) cannot manage any more. They decided to hire a domestic helper even though their father strongly rejected this idea.

The relationships between G2 and G1 are close, because G2 make decisions together and share information by using their WeChat conversation group. For example, G2-D1 took the responsibility to search for a helper and communicates with the nursing home that has supplied helpers. She takes responsibility for the bank cards of their parents’ account and photographs every receipt of what is spent, sharing this on the family WeChat group.

The three sisters (G2) tried to protect their father’s feelings about hiring a helper and told him that the helper is a distant relative, knowing that their father would feel more comfortable in asking for her help.

#### The Zhao family

The Zhao family is made up of three generations: Mrs. G1, three daughters in G2, and three only children in G3 (see Figure 5.9 below). Their family gatekeeper (who the researcher first caught up with) was G2-D1’s husband, who was one of the participants in the focus group. After this conversation, G2-D1 showed much more interest in this study and actively contacted the author.


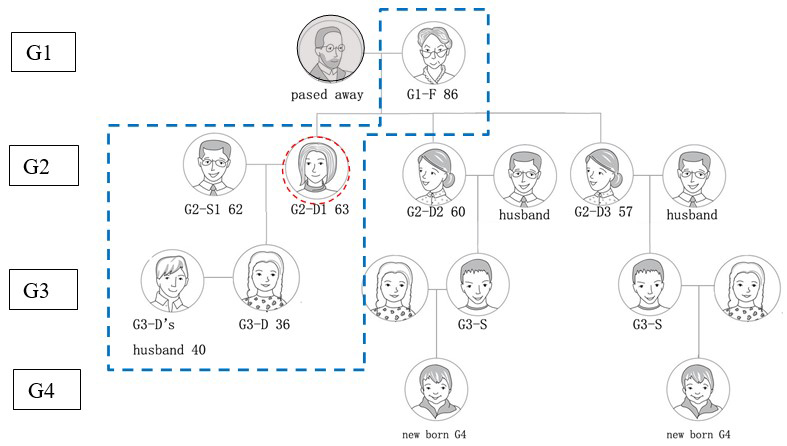


Figure 0.9: Zhao family

As a result, follow-up talks were held at the home of the Zhao family with G2-D1. Interviews with G2 and G3 were performed face to face, while a visual call was made to speak with G1. Mrs. G1 is already 84 years old, and lives with a live-in care worker now, but she is in generally good health.

There are three daughters in G2, but two of them have moved and settled down in Tianjin. In their family, G2-D3 used to take the responsibility of looking after their mother because they lived close to each other in the city of Qiqihar. However, the situation changed when G2-D3 tried to extend her business to Yunnan province in the southeast area of China. G2-D1 persuaded her mother (G1) to come and live with her to overcome this, which she did, but after a year, Mrs. G1 refused to stay, demanding that she return to her own home.

#### The Wang family

The Wang family is made up of four generations: the G1 couple, two sons and two daughters in G2, four only children in G3, and a two-year-old girl in G4 who is G2-D3’s granddaughter (see Figure 5.10 below). The interviews with the Wang family concentrated on the G1 couple via video phone call, while G2-S1, his wife and his daughter (G3) were interviewed face to face.


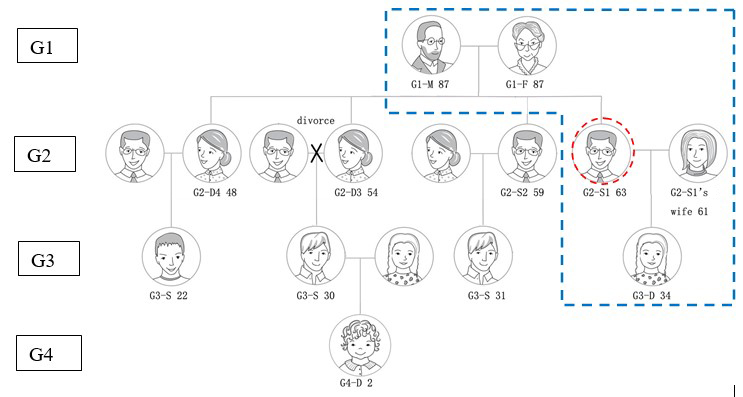


Figure 0.10: Wang family

Three of G3 live in Shenyang (in Liaoning province in the northeast of China), while their grandparents (G1) and G2-S1 live in Tianjin. G1 live in a relatively new commercial apartment that was bought by G2-D3. In turn, G1 asked G2-S2 to sell their old apartment to fund their later-life expenses.

The G1 couple needed help during this interview period. Mrs. G1 was so frail that she had gone to the hospital emergency room twice during 2019. Thus, the children (G2) tried to hire a helper who could live with the G1 couple to meet their daily needs and deliver timely notifications of problems. However, they changed helpers frequently within the year, and it has been a real headache to find a long-term live-in maid.

### Families who have no G1

This group of three families, unlike the other two groups, have simple family arrangements: they have lost G1, leaving G2 as the oldest generation in their families and are potentially reliant on only one child.

#### The Kong family

The Kong family is made up of three generations: the G2 couple, the G3 couple (the only-child generation), and two children in G4 (see Figure 5.11 below). They (except G3-husband’s father), G3-D and her husband, G3-D’s parents and her children are co-resident in G3’s three-room apartment now. G3 are a new middle-class household in this family.


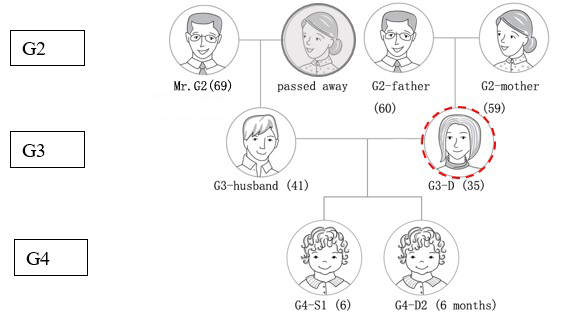


Figure 0.11: Kong family

Here it may be seen that the family structure is not the typical structure of a ‘4-2-1’ family. Their family structure is a ‘3-2-2’ type. G3 are the backbone generation in this family. G2 (G3-D’s parents) in this family belong to the working class: their jobs are worker and salesperson, and their education level is no higher than the high-school diploma. They (G3-D’s parents) sold their only property when G3-D asked them to live together.

G3-D’s husband has only a father (G2) who lives alone in the countryside in Shanxi province. His mother passed away more than ten years ago. His father is a farmer and plants date palms for a living. The G3 couple takes the G4 children to visit the father once a year.

The G3 couple were classmates during their postgraduate degree. They supported each other when G3-D’s husband lost his mother in that period. One may easily believe it when G3-D says that she and her husband have a solid foundation to their relationship. Her husband (G3-S) is an engineer for the national railway company and has to travel for his projects, but she has never worried about their relationship even though they may only meet once a month.

There are many lifestyle differences between G2 and G3, and co-residence makes the tensions between them apparent. It is mostly seen in that G2 asks for G3’s support directly, and in G3’s expectations of caregiving from her parents to their children, but obviously, in return, G3-wife cannot receive ideal support, which may lead to negative effects on their family practice.

#### The Han family

The Han family is made up of three generations, including a G2 couple, a G3 couple and a son in G4 (see Figure 5.12 below). The three generations (G3-husband’s parents, G3-husband and G3-wife, and their son G4-S) are co-resident in G3’s two-room apartment in Tianjin.


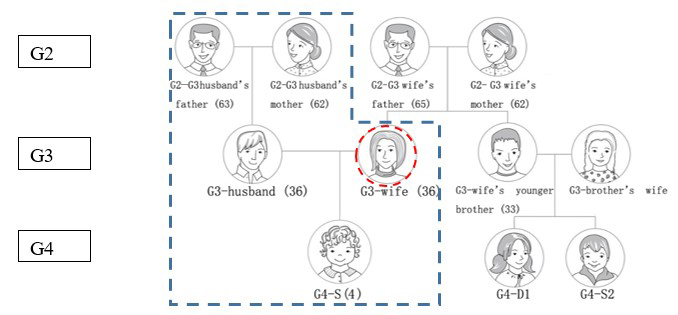


Figure 0.12: Han family

In the Han family, G3-husband’s parents were civil servants in Anshan city (a tier-three city in the northeast of China), Liaoning province. They are believed to be of middle-class status in their hometown; such is their pension, occupation and education level. They (the G2 couple) live together with G3 in G3’s apartment in Tianjin because G4 needs to be looked after since G3-D1 went back to work. G2 still keep their apartment in Anshan city for more choices in later life.

Here, some differences have to be addressed. It can be seen from this table that in G3-wife’s family, there are (unusually) two children in their family, which means that G3-wife has a younger brother who shares the duty of caring for their parents (G2). Their parents depend on their son (G3-younger brother). Their parents (G2) were farmers at first, who could have a second child if the first child were a girl, but it is difficult to earn a living through farming and also to support two children going to college. Then, the G2-couple (G3-wife’s parents) started their own business of selling fruit. After a car accident, G3-wife’s mother became disabled, and caregiving is provided by her husband. Now, her parents (G2) live close to their son (G3-younger brother) in separate apartments that were exchanged for their original house and courtyard under the background of urbanisation.

#### The Fu family

The Fu family is made up of three-generation: a G2 couple, a G3 couple and a girl in G4 (see Figure 5.13 below). The G2 couple (G3-S’s parents) moved to Qingdao to help G3 take care of G4 since their retirement. The interviews focus on G3-husband’s parents and the G3 couple in their family.


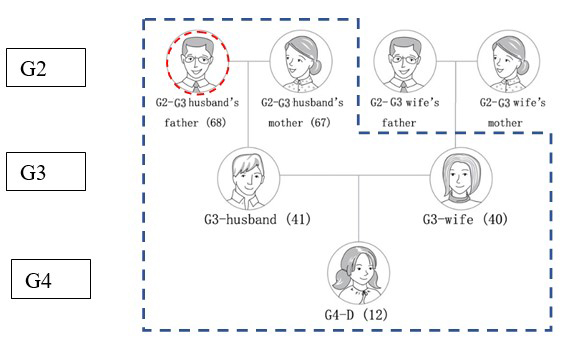


Figure 0.13: Fu family

There is a typical ‘4-2-1’ family structure in the Fu family. Their G2, namely G3-S’s parents, are located in the middle social status layer, evidenced by their occupations and pension; also, they own two properties in Tianjin. After they moved to Qingdao city, they sold one of their apartments in Tianjin to purchase a good school district apartment that benefits their granddaughter (G4). Now, they keep one apartment in Tianjin in hand and live in the school district apartment to look after G4.

The G3 couple are classmates from the Architecture School of Qingdao University. They settled down there after graduation and earn sufficient money to have a middle-class lifestyle. They bought an apartment, but it is outside the school district. Their busy working life occupies their time, leaving them without the energy to take care of G4. Therefore, G3-S’s parents (G2) decided to provide support after they retired.

## Summary

This chapter aims to situate the fieldwork chapters in context. It has given a simple illustration of the history and living standards of Tianjin. The chapter has also introduced each of the families that will be discussed next. It introduced these families in terms of their backgrounds and their relationships. To symbolize the family members of the interviewee, their family tree has been illustrated by cartoon drawings. I have adopted this approach because it allows the reader to become familiar with these families first, before we jump into their family stories more specifically.

## References

Baars, J. and Dohmen, J. (eds.) (2013) *Ageing, meaning and social structure: Connecting critical and humanistic gerontology*. Policy Press.

BBC NEWS (2018) 'China birth rate: Mothers, your country needs you!',edn), 25 December 2018. [Online] Available at: <https://www.bbc.co.uk/news/world-asia-china-46558562> (Accessed: April, 2020).

Breitung, W. (2012) 'Enclave urbanism in China: Attitudes towards gated communities in Guangzhou', *Urban Geography*, 33(2), pp. 278-294.

Deloitte Global (2017) *White Paper on Environment for Investment in Tianjin Economic-Technological Development Area*. [Online]. Available at: <https://www2.deloitte.com/content/dam/Deloitte/us/Documents/about-deloitte/us-mfg-tianjin-economic-technological-development-area.pdf> (Accessed: 6 January 2021).

Feng, D. (2018) 'Suzhou, Beijing and Tianjin Rank as China's Most Livable Cities on EIU Global List',edn), 15 August 2018. [Online] Available at: <https://www.yicaiglobal.com/news/suzhou-beijing-and-tianjin-rank-as-china-most-livable-cities-on-eiu-global-list> (Accessed: October 2020).

HKTDC Research (2020) *(Data and Profiles--Mainland China Provinces and Cities--Municipalities) Tianjin: Market Profile*. Available at: <https://research.hktdc.com/en/data-and-profiles/mcpc/municipalities/tianjin> (Accessed: 6 January 2021).

Kiprop, V. (2020) *Fertility Rates In China: 1930 To 2020*. Available at: <https://www.worldatlas.com/articles/fertility-rates-in-china-1930-to-2020.html>.

Liu, W., Zhang, J., Yang, S., Wang, X. and Huang, Z. (2018) ' Jing-jin-ji di qu yang lao di chan xiang mu diao yan he yang lao di chan fa zhan jian yi (Research on senior care real estate projects in the Beijing-Tianjin-Hebei region and suggestions for senior real estate development)', *shang ye jing ji (Business economy)*, (1), pp. 32-34.

Liu, Z. and Cao, H. (2017) 'Spatio-temporal urban social landscape transformation in pre-new-urbanization era of Tianjin, China', *Environment and Planning B: Urban Analytics and City Science*, 44(3), pp. 398-424 [Online] DOI: <https://doi.org/10.1177%2F0265813516637606> (Accessed: August 2019).

Mu, X. (2020) *Ten pictures to understand the development status of China's new first-tier cities*. Available at: <https://www.qianzhan.com/analyst/detail/220/200302-0425b0df.html> (Accessed: November 2020).

Tianjin Civil Affairs Bureau (2018) *Regulations on the Promotion of Old-Age Services in Tianjin (Tianjin shi yang lao fu wu cu jin tiao li)*. [Online]. Available at: <http://mz.tj.gov.cn/ZWGK5878/ZCFG9602/DFXFG1195/202008/t20200805_3370652.html> (Accessed: August 2020).

Tianjin Civil Affairs Bureau (2019) *Interpretation of the Three-year Action Plan of Tianjin Municipality for the Promotion of the Development of Elderly Care Services (2019-2021)*. Available at: <http://www.tj.gov.cn/zwgk/zcjd/202005/t20200519_2376834.html> (Accessed: July 2020).

Tianjin Local History Compilation Committee Office (2005) 'Natural geographical environment', in *GENERAL HISTORY OF CHINA: TIANJIN*. 中国青年出版社(CHINA YOUTH PUBLISHING GROUP).

Tianjin Local History Revision Committee (2015) *Construction of China (Tianjin) Pilot Free Trade Zone*. Available at: <http://www.tjsdfz.org.cn/tjsq/zmqjs/> (Accessed: September 2020).

Tianjin Municipal People's Government (2016) *Pension Situation in 2016 of Tianjin*. [Online]. Available at: <http://www.tj.gov.cn/xw/bum/201708/t20170811_3610352.html> (Accessed: October 2020).

Tianjin Statistical Bureau (2019) *Statistical Report of Tianjin Economic and Development in 2018*. [Online]. Available at: <http://www.tj.gov.cn/tj/tjgb/201903/t20190311_3650936.html> (Accessed: October 2020).

Xie, Y. (2013) *Gender and family in contemporary China* (13-808). University of Michigan: Social Research. [Online]. Available at: <http://citeseerx.ist.psu.edu/viewdoc/download?doi=10.1.1.405.7974&rep=rep1&type=pdf> (Accessed: September 2020).

Xin Hua News (2019) 'Xinhua Headlines: "Jing-jin-ji": China's regional city cluster takes shape', *Xin Hua News*edn), 25 Feburary 2019. [Online] Available at: <http://www.xinhuanet.com/english/2019-02/25/c_137849309.htm> (Accessed: October 2020).

1. The concept of livability is simple: it assesses which locations around the world provide the best or the worst living conditions. (resource from <https://www.eiu.com/topic/liveability> ). [↑](#footnote-ref-1)
2. CNY: Chinese Yuan [↑](#footnote-ref-2)
