# Supplementary material for: Shifting elder-care practices in Chinese middle-class families
Source: PLoS One. 2023 Mar 24;18(3):e0283533. doi: 10.1371/journal.pone.0283533 (PMC10038285; doi:10.1371/journal.pone.0283533)
Supplement: Attachment — Submitted filename: cover letter 1030 Response to editor and reviewer.docx [file pone.0283533.s004.docx]

**Dear editor:**

Can we thank you and the reviewer for their attention to this paper that has provided us with the opportunity to strengthen the submission. We did not upload the manuscript with tracing marks, because our manuscript has a major revision and three authors all contributed on it, so we did not save the version with tracing marks. However, we did do the revision by following the reviewer’s suggestions.

To response point to point below:

1. The abstract has been rewritten in line with the advice (page 1)
2. The introduction has been radically shortened and tightened to give (we hope) a clearer line of argument. In each paragraph we have set out the situation as discussed in the literature and have posed questions that need to be addressed as a result. (pages 2-5)
3. The Methods section and new data analysis section have also been recast and are less discursive in style. They make more clear the imperative behind research approaches. (pages 5-9)
4. Relatively unfamiliar Chinese terms such as *hukou* have been translated for the benefit of the reader. (page 6)
5. The question of what percentage of participants who refused recording has been addressed. (page 8)
6. While the field work was undertaken by the first author all subsequent analysis was informed by discussion between the three authors which is explained. (page 9)
7. The topic guide is provided (appendix 1).
8. There is greater clarity about the mechanics of the interviews (page 7)
9. More discursive discussion of the family types is taken into the discussion (pages 20- 25)
10. Resources means resources that families may deploy– occupation, education level, income/wealth and property ownership). To avoid confusion e.g., table 4 (page 19) we have labelled respondents and broken down the resources into elements.
11. The discussion has been reworked in line with advice and includes a section setting out the limitations.
12. The second and third authors who are native British English speakers took a greater role in reworking the paper and its language.

Warm regards

Lu, Rose and Andy
