# Supplementary material for: Shifting elder-care practices in Chinese middle-class families
Source: PLoS One. 2023 Mar 24;18(3):e0283533. doi: 10.1371/journal.pone.0283533 (PMC10038285; doi:10.1371/journal.pone.0283533)
Supplement: Attachment — Submitted filename: Response to editor and reviewer 27-02-23.docx [file pone.0283533.s005.docx]

**Dear editor:**

Can we thank you and the reviewers for your attention to this paper that has provided us with the opportunity to strengthen the submission.

**Reviewer 1**: Some final check on grammar, wording, editing is still necessary (example: "eldercare" versus "elder care" (with or without space), but better seems "elderly care" and more preferably another term like "care for older persons"; page 4: no space between -2 and parents); no first names in author referencing; et al. yes or not in cursive)

**Responses:** We have used elder-care consistently through the paper in line with the suggestions. The word `elderly` is seen by gerontologists as othering and is not used.

**Page 4:** Spaces have been reduced between ‘-2’ and ‘parents’.

The reference style followed is that set out in the PloS template.

**Reviewer 2**:

| **Comments** | **Responses** |
| --- | --- |
| 1. Abstract  - The abstract could be written more in-line with a typical research abstract (background, purpose/aims, methods, results, discussion/conclusion). As written, it is unclear the underlying methods/rigor of this study or how the points the authors refer to were identified.  - There remains grammatical errors in the abstract. | The abstract has been re-written with clearer progression between these points.  Grammatical issues have been addressed |
| 2. Introduction  - Second paragraph, second sentence. I’m not sure this sentence is asking a question.  - While interesting, the introduction can be shortened considerably. At present, it is more than 1600 words in length. In contrast, the typical medical journal manuscript is typically 3000-3500 words total. | We have omitted the question mark  We have shortened the introduction. The reviewer makes comparison with medical journal manuscripts, but our view is that while it is wise to be succinct, sociological studies demand more grounding and particularly, in the case of studies in other countries, with which the reader may have little familiarity |
| 3. Methods  a- Consider shortening. This reads much more like a dissertation rather than a journal article.  b- Consider using headings (e.g., sampling strategy, data analysis)  c- Consider reporting methods in line with qualitative reporting guidelines (e.g., COREQ)  d- The authors could consider removing several unnecessary details in the methods (i.e., gifts, small favours etc.)  e- Discussion of methodological issues of the study (e.g., the discussion of why respondents preferred to not be recorded) belong in the discussion section, not methods.  f- Paragraph under the image 1 belongs in discussion other than the description of IRB approval.  g- More details regarding the qualitative methods are needed. How many people coded? Were transcripts professionally transcribed? What analytic method did the authors use? How did the authors code and develop themes? Did they reach data saturation? How was the interview guide developed? Refined? How were codes developed and refined? Please follow qualitative reporting guidelines as suggested above.  h- Typically names of families, patients, etc. are not included in manuscripts due to concerns for HIPAA and the need to de-identify respondents. Consider changing names to abbreviations (e.g., the “H Family” or “family # 1”). Given financial information is also described in this paper (Table 4 resources of the Han family) I would strongly urge the authors to anonymize respondents. | a) We have shortened this section b) We have inserted more headings to guide the reader c) In line with the COREQ reporting checklist we have inserted more description of research items. d) The issues that the reviewers feel are unnecessary have been deleted. e) and f) We disagree with the assessment that hesitancy about voice recording belongs in the discussion and have retained this as a methodological issue. g) Each interview was transcribed and translated by author 1 though the final thesis was proofread by a native British English speaker. All interviewees were sent a copy of their transcript to review and edit if they so wished. An English language version was then stored in NVIVO. The transcripts and research were shared with the second and third authors who discussed the framework for analysis.   Definitions of data saturation vary but our view was that it was reached when no further codes could be generated.  h). Thank you for the suggestion that names should be replaced by numerical identifiers or initials only. Can we assure the reviewers that no actual family names were used. Family names that were not present among the participants were randomly assigned. In our view the use of names preserves the human connection between the reader and the families in the study. The context may be different, but many families face these issues. |
| 4. Findings  - In this section only report the findings from the study. Any discussion/expansion of the findings should be included in the discussion section. | We have followed the guidance and more discursive thoughts are now in the discussion |
| 5. Discussion  - Lacking a limitations paragraph | The paragraph of limitations is on the page 25-26. |

Dr Lu Wang

Professor Rose Gilroy

Dr Andy Law
